# Assessment of the Impact of Technical Incidents in Critical Situations Using High-Fidelity Simulation Techniques in Pediatric Intensive Care

**DOI:** 10.7759/cureus.64381

**Published:** 2024-07-12

**Authors:** Corentin Biot, Ismail Sanoussi, Yoann Marechal

**Affiliations:** 1 Pediatrics, CHU Charleroi-Chimay, Charleroi, BEL; 2 Neonatology, CHU Charleroi-Chimay, Charleroi, BEL

**Keywords:** checklist approach, ventilated patients, technical incident, pediatrics and neonatology, high fidelity simulation training

## Abstract

Introduction

In certain fields such as anesthesia and critical care, technical incidents are rare events; however, when they occur, they disrupt workflow, optimal patient care, and survival, with human factors often implicated. In pediatric resuscitation, the impact of these incidents on patient care has not yet been thoroughly explored through simulation. Consequently, we investigated how healthcare teams integrate technical incidents in critical situations and whether this interferes with the adequate management of patients.

Materials & methods

In a single-blind randomized study utilizing high-fidelity simulation, we incorporated a pediatric scenario involving hypoxemia in an intubated and ventilated infant where the endotracheal tube (ETT) was obstructed. A technical incident (disconnected oxygen supply) was either present (TI+) or absent (TI-) in the scenario. We compared reaction times for "removal of the obstructed ETT" between the two groups (TI+ and TI-). Additionally, we recorded and analyzed reaction times for "bag ventilation" and "repair of the technical incident" in the TI+ group. To assess the scenario's credibility, we conducted an analysis comparing the medians of evaluation forms that were anonymously completed by participants at the end of the sessions.

Results

In total, 10 simulation sessions were conducted, five TI+ and five TI-. The time required for removal of the obstructed ETT in the presence of a technical incident was significantly prolonged compared to controls (Mann-Whitney test, *p*=0.03). Furthermore, bag ventilation precedes tube removal in the TI+ group, a contrast to the TI- group, which quickly removes the obstructed ETT before stabilizing the patient with bag-mask ventilation.

Conclusion

Technical incidents in simulated pediatric scenario adversely affect urgent care in ventilated children. Developing and validating a procedural response to these situations through further simulation is imperative.

## Introduction

Rare occurrences of technical incidents can significantly compromise patient care and escalate morbidity or mortality rates [1]. These incidents, arising from equipment malfunction, material failures, or human errors, underscore the importance of addressing the human factors associated with the manufacturing and maintenance of medical equipment as a key preventative measure [2,3]. Experts in human factors and ergonomics focus on understanding the interplay between humans and systems to enhance system performance, human efficiency, user satisfaction, and ultimately patient safety. However, these developments are sometimes implemented by designers without fully integrating the medical reasoning and clinical considerations essential for effective patient care [4].

Human performance, a concept extensively explored in aviation, refers to the execution of physical or mental tasks influenced by both intrinsic and extrinsic factors [5]. Parameters, such as speed, accuracy, and attentional demand, are crucial metrics for assessing human performance, with greater speed, higher accuracy, and lower attentional demand representing optimal standards [6,7].

In the realm of anesthesia, where technical incidents are infrequent yet impactful, healthcare teams often lack adequate training to manage such situations effectively. Routine equipment checks may not be consistently performed, leaving room for improvement in incident management. Cognitive aids, like checklists and decision algorithms, coupled with simulation training, have demonstrated efficacy in enhancing incident management [8,9]. However, research in the pediatric context is notably lacking, particularly in evaluating the impact of technical incidents in critical situations through simulation.

This prospective study aims to fill this gap by investigating how healthcare teams respond to technical incidents during critical scenarios using high-fidelity simulation at the Simulation Center of Marie Curie Civil Hospital (Charleroi, Belgium). The study seeks to evaluate whether these incidents may impede adequate patient care and, based on the findings, suggests the development of procedural guidelines to mitigate the impact of such incidents on patient treatment, thereby enhancing the response times and overall patient outcomes.

## Materials and methods

**Figure 1:**
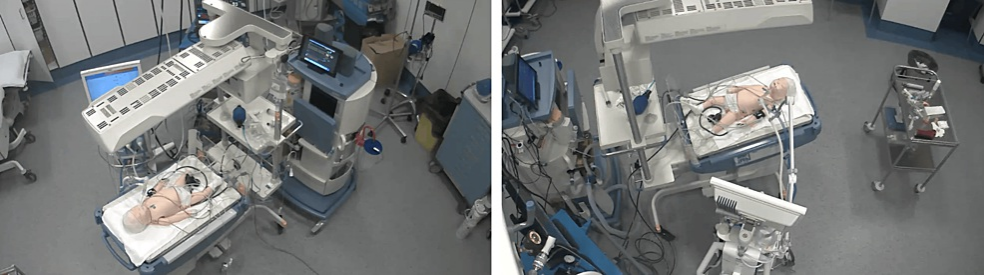
General view of the simulation room and its equipment

**Figure 2:**
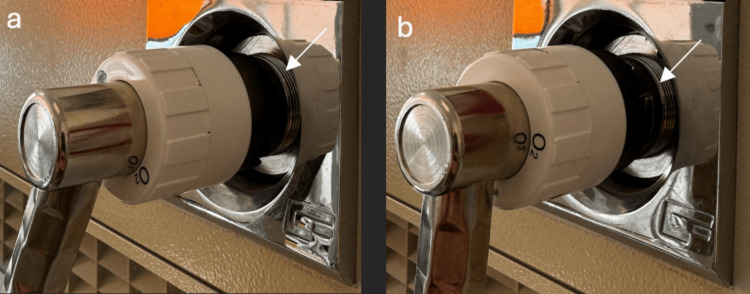
Technical incident: (a) Absent, (b) Present.

**Figure 3:**
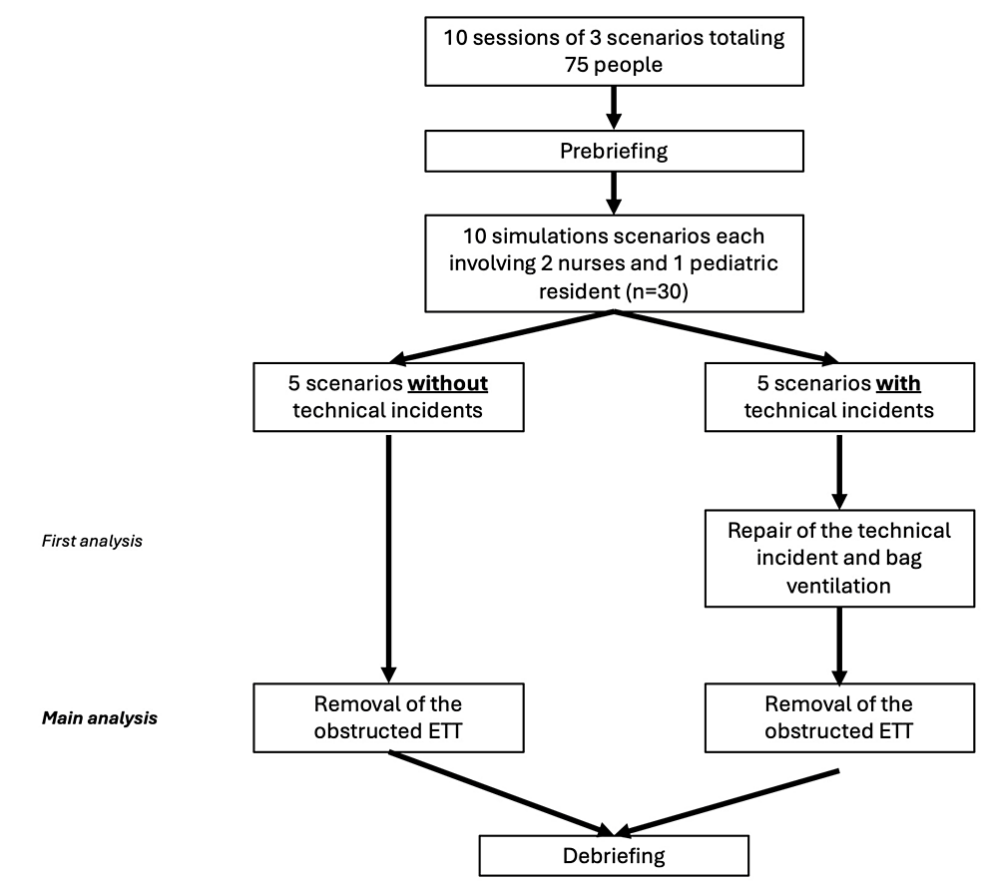
Flow Chart ETT: Endotracheal tube

The simulation scenario focuses on managing hypoxemia in an intubated and ventilated infant with an obstructed endotracheal tube (ETT) and arterial and venous catheters in place, along with continuous cardiorespiratory monitoring (HR, RR, SpO2, BP, EtCO2). The simulation sessions take place in a simulation center, using the same PICU environment as in the working unit including the ventilator Draeger VN500 (Dräger, Lübeck, Germany) and the reanimation table Giraffe GE Healthcare (Chicago, IL, USA) (Figure 1). In this single-blind 1:1 randomized study, two types of simulation sessions were conducted: one without the technical incident (TI-) and one with the technical incident (TI+). The technical incident involved a connection failure of the oxygen supply to the ventilator, which was handled by personnel responsible for equipment preparation and verification (Figure 2). The time taken for "removal of the obstructed endotracheal tube" was systematically recorded during each simulation for both groups (TI- and TI+). For the TI+ group, two additional measurements were taken: "repair of the technical incident" and "bag ventilation" (see flow chart, Figure 3).

To ensure learner safety, the mannequin "does not die" during the scenario and the facilitator intervenes at 5 minutes to guide learners towards appropriate management, particularly addressing the endotracheal tube obstruction and potentially reviewing the technical problem with the ventilator with one of the learners to address the problem within maximum 60 further seconds. The facilitator was a senior in neonatal intensive care medicine, aware of the scenario and the presence or absence of the technical incident. The time for a new intubation was not studied as it was not considered a technical objective in the scenario.

Each scenario lasts approximately 10 minutes, with participants playing their designated roles to streamline debriefings regarding non-technical session objectives. One pediatric resident in his first or second year of training and two experienced intensive pediatric nurses worked together on the scenario.

Simulation sessions begin with a two-part prebriefing: a meeting session outlining simulation objectives and rules, followed by a simulation room session are presented, and certain technical procedures are reviewed upon request. The introduction is followed by a sequence of three scenarios with different technical and non-technical objectives which are debriefed each time. The session concludes with a general summary and the identification of areas of improvement. To eliminate potential confounding factors resulting from participation in a first simulation session, we decided to place the studied scenario second or third in the half-day session. After the simulation sessions, participants were asked to complete a satisfaction form in which the realism of the scenarios was evaluated.

For the statistical analysis, GraphPad Prism software (10.2.1) has been employed to input and analyze reaction times and the credibility of compared scenarios. This analysis utilized a non-parametric Mann-Whitney test.

## Results

A total of 10 simulation sessions were conducted: five each for the TI- group and five for the TI+ group, resulting in a dataset comprising ten reaction times for the variable "removal of the obstructed tube" and five for the variables "repair of the technical incident" and "bag ventilation." Each scenario involved two nurses and one pediatric resident, totaling 30 key participants. Additionally, 45 nurses were present during the half-day sessions and participated in other scenarios, but also contributed their evaluations as observer on the credibility of the scenario.

**Figure 4:**
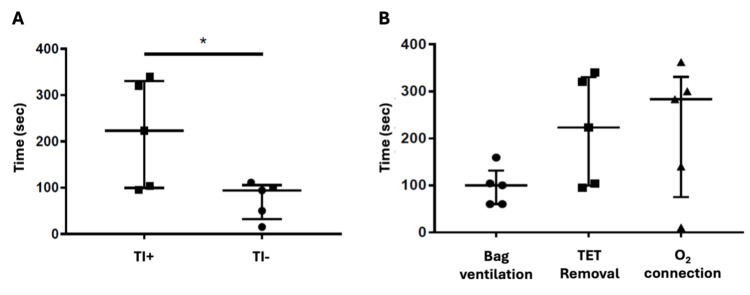
(A) Comparison of time (sec) for removal of obstructed endotracheal tube in case of simultaneous technical incident (TI+) or not (TI-), *p=0.03; (B) Time (sec) to try to resolve the situation in case of IT+

Comparing the time required for the removal of an obstructed ETT with or without a concurrent technical incident, a notable increase in reaction time was observed in scenarios featuring a technical incident (Figure 4A). Specifically, in scenarios without technical incidents, the median time for tube removal was 94 seconds (minimum 15 and maximum 111 seconds), while in scenarios with technical issues related to the ventilator, it was 223 seconds (minimum 95 and maximum 340 seconds; *p*=0.03). Interestingly, two teams in the TI- group followed the DOPES acronym [10] and identified the obstructed ETT by quickly suctioning through the tube before bag ventilation. They started bag and mask ventilation after the removal of ETT. This observation has never been made in the TI+ group. Furthermore, we noted that in the presence of a concurrent technical event, bag ventilation occurred before endotracheal tube removal (Figure 4B), despite the airway obstruction alarm being present alternatively with the fluid connection alarm on the ventilator. We can also see that in the TI+ group, the resolution time for the technical incident (oxygen connection) is not significantly different from the resolution time for the ETT obstruction issue, suggesting that there is no prioritization of tasks.

The feedback in the satisfaction form completed at the end of the session did not indicate a significant perceived difference of clinical realism when a technical incident was introduced, suggesting that the inclusion of such incidents may occur in real life (data not shown).

## Discussion

The aim of this study was to assess healthcare professionals’ management of hypoxemia acquired during invasive ventilation in children, especially when technical incidents complicate the situation, using high-fidelity simulation. Like observations in other fields where human factors can contribute to or exacerbate issues in care delivery, pediatric resuscitation is also influenced by human factors. The optimal response is prompt removal of a completely obstructed endotracheal tube. However, the presence of a technical incident significantly delays the time of the correct action, thereby compromising the survival of critical patients.

In scenarios without technical incidents, participants swiftly removed the endotracheal tube and proceeded to ventilate the mannequin with a bag and mask, focusing on clinical aspects and patient management - a desirable response. Nevertheless, in scenarios featuring a technical incident, we observed a fixation error [6], where participants concentrated on reconnecting fluids and quickly switched to bag ventilation over the obstructed endotracheal tube without recognizing the ineffectiveness of their actions. Their attention, which should have been focused on patient care and ventilation monitoring, was diverted to managing the ventilator.

The primary limitations of the study stem from the small sample size and the fact that the simulation was conducted in a dedicated room rather than in situ, within the participants’ usual care environment. While the current setup allows learners to acquire skills and review their practices, it may lead to less detailed incident analysis than what might occur in their regular work settings, potentially affecting reaction times for technical incidents and prolonging reaction times for learners less familiar with the equipment used. In situ simulations, which increase perceived realism among participants and validate procedures [11], could disrupt clinical work if conducted without additional personnel on session days, especially during high workload periods, increasing the risk of incidents [12].

Another limitation could be the participants' level of training, in our case, first- and second-year residents. Although they are subject to the same human factors (e.g., tunnel effect), they may be more susceptible to them than their more experienced colleagues.

Simulation positively impacts patient safety by facilitating the learning of technical procedures, the acquisition of non-technical skills, and bridging the gap between theory and practice [13,14]. Furthermore, navigating scenarios involving technical incidents has a significant ethical advantage and is supported by a comprehensive three-stage debriefing process, which has been shown to improve incident management. Debriefing is crucial for maintaining a pedagogical framework and acquiring knowledge during simulation sessions, with regular repetition of simulations reinforcing the knowledge gained.

We have explored the tools and strategies to mitigate the consequences of technical incidents and improve response times in pediatric resuscitation. Cognitive aids have proven effective in enhancing patient management and survival rates in anesthesia. The Society for Pediatric Anesthesia Committee has developed checklists and cognitive tools to guide response and management during critical events, serve as informational resources, and facilitate understanding of necessary actions in critical situations [8].

Errors related to equipment preparation and usage, documented in pediatric intensive care literature, include ventilator use and preparation errors, such as gas failures from disconnected or incorrectly connected cables, and non-invasive blood pressure measurement errors, like using an inappropriate cuff size or incorrectly connecting cables to the monitor [15,16]. Other errors involve mask ventilation, often due to an inappropriate mask size, and intubation errors, like using incorrect endotracheal tube and laryngoscope blade sizes for the patient’s age and weight [17]. Our findings, based on simulation session observations and frequently encountered errors, have led to the development of a procedural protocol to guide responses when technical incidents occur with pediatric ventilators during resuscitation efforts.

Rigorous equipment and material checks by healthcare personnel are crucial for mitigating the risk of technical incidents and subsequently reducing morbidity and mortality rates. Based on this work, we suggest implementing a clinical approach directly into the contextual help menus of ventilators using existing cognitive aids (such as DOPES or others), as a checklist like this could assist healthcare providers in prioritizing tasks during such situations. We recommend measuring their effectiveness in simulation before clinical implementation to reduce cognitive load on clinicians and the risk of delayed management. In the era of artificial intelligence, integrating such cognitive aids directly into the ventilator’s help menu could be beneficial [18], although collaborations with different industries have not yet been agreed upon.

## Conclusions

Technical incidents present a significant risk in intensive care environments, potentially culminating in fatal errors in patient management. Our study, utilizing high-fidelity simulation, has underscored the adverse impacts of these incidents on the prompt provision of critical care for ventilated children. The validation of solutions such as cognitive aids or procedural checklists, through simulation, holds significant potential for mitigating risks associated with these incidents and enhancing management practices. Therefore, identifying a partner firm to help implement these checklists and conducting field trials in real-world settings are crucial next steps to ensure the effectiveness and reliability of these tools in clinical practice.
